# Family’s presence associated with increased physical activity in patients with acute stroke: an observational study

**DOI:** 10.1590/bjpt-rbf.2014.0172

**Published:** 2016-06-16

**Authors:** V. Prakash, Manushi A. Shah, K. Hariohm

**Affiliations:** 1 Ashok & Rita Patel Institute of Physiotherapy, Charotar University of Science and Technology, Changa, Gujarat, India; 2 Intern, Ashok & Rita Patel Institute of Physiotherapy, Charotar University of Science and Technology, Changa, Gujarat, India; 3 Clinical Physiotherapist, Spring Physiotherapy Centre, Chennai, Tamilnadu, India

**Keywords:** family support, acute stroke, physical activity status, time spent alone

## Abstract

**Background:**

Inherent differences in organization of stroke care and rehabilitation practices in various settings influence the activity levels of patients in the hospital. The majority of published studies have been carried out in developed countries such as the United States, United Kingdom, Australia, Switzerland and Belgium; however, data from developing countries are scarce.

**Objective:**

To measure the amount and nature of physical activity of patients admitted to medical wards of Indian hospitals and to assess the association between family presence and the patient and between the patient’s functional status and their physical activity level.

**Method:**

This is an observational behavioral mapping study. A trained physical therapist recorded the patients’ (N=47) physical activity level through direct observation in the ward using a predetermined observation scheme.

**Results:**

Participants were found inactive and alone for 19% (inter quartile range [IQR] 12-36%) and 15% (IQR 10-19%) of the time during the day, respectively. They spent 46% (IQR 31-55%) of the time in therapeutic activities and 31% (IQR 22-34%) of the time in non-therapeutic activities. The family was present with patients 50% of the time during the day. Family presence with the patient and the patient’s moderate dependence in daily activities are positively associated with their activity levels.

**Conclusion:**

Patients with stroke admitted to Indian hospitals spent less time being inactive and alone and more time with family participating in therapeutic activities. The presence of family members with the patients during hospital stay may be a significant resource for encouraging patients to be more active.

## BULLET POINTS

- The characteristics of the stroke rehabilitation setting can influence physical activity levels.
- Stroke patients in India are more active and spend less time alone.
- Family presence is associated with a higher physical activity level in patients after acute stroke.

## Introduction

Increased amount of physical activity in the early phase after stroke can promote positive outcomes in the long term^1^ ^,^ ^2^. A recent review on physical activity patterns of hospitalized stroke patients reported that patients spent most of their time alone and spent less time in moderate-to-high level physical activity, such as sitting unsupported, standing, and walking (median 21.0%, interquartile range [IQR]12.8% to 27.7%)^3^. Further, there are wide variations in patients’ activity levels during acute stage of stroke across different settings^4^ ^-^ ^6^. Understanding what drives these discrepancies could help us to improve stroke care and early rehabilitation^7^.

Several factors were identified as sources of variation in physical activity levels observed in the acute stage of stroke^3^ ^,^ ^5^ ^,^ ^8^. Patients with severe dependence in functional activities spent more time inactive and alone (90% of the day) in bed; organized stroke care (stroke units) and longer therapy time, especially time involved in autonomous practice encouraged patients to be more active than conventional care^3^ ^,^ ^5^. In addition to patient-related factors, differences in hospital policies (i.e., nurse-patient and therapist-patient ratio, restrictions on manual handling of patients and access to common recreation areas in hospitals) have been found to influence the physical activity patterns of patients after acute stroke^5^ ^,^ ^8^ ^,^ ^9^. The majority of published studies were carried out in developed countries such as the United States, United Kingdom, Australia, Switzerland, and Belgium^3^; however, data from developing countries are scarce. Stroke is one of the leading causes of disability in India^10^ ^-^ ^12^. There are no published data available on the patterns and influencing factors of physical activity in patients with stroke admitted to Indian hospitals.

Stroke care in India is usually delivered in medical wards as only few stroke units exist^10^. Many of these wards require a family member to remain with patients during their hospital stay. In the acute stage after stroke, therapy is usually provided at the ward, which is not structured for providing exercise. To account for these differences in developing optimal acute stroke rehabilitation care in India, data on physical activity patterns and their influencing factors are necessary.

Therefore, the objectives of this study were (1) to quantify the amount and nature of physical activity of patients admitted to the medical wards of Indian hospitals and (2) to assess its association with patients’ functional status and presence of family members.

## Method

### Study design and data collection

This is an observational behavioral mapping study. Ethical approval was obtained from the Institutional Review Board of Ashok & Rita Patel Institute of Physiotherapy, Changa, Gujarat, India (protocol approval number: ARIP/IRB/14/23). Before the onset of the study, the protocol was discussed and approved by the medical superintendents of all three participating hospitals. All patients were informed about the study protocol and informed consent was obtained from each participant.

### Study participants and Setting

Our study participants included acute stroke patients (within 14 days after onset) managed in medical wards in three private hospitals located in Vadodara city, Gujarat, India. Data were collected between June and October 2014. We used the World Health Organization’s definition of stroke^11^ ^:114^ as “rapidly developing clinical signs of focal or global disturbance of cerebral function, with symptoms lasting 24 hours or longer or leading to death, with no apparent cause other than that of vascular origin”. Participants were patients diagnosed as having stroke by the treating physician, who were medically stable and did not require acute medical intervention. The primary purpose of their ongoing hospitalization was for rehabilitation.

All patients diagnosed with stroke (n=53) were approached, and 51 (96.0%) agreed to participate in the study; two refused to participate for unknown reasons. Forty-seven patients met the inclusion criteria because four were found to have had a stroke longer than 14 days. The patients’ characteristics are described in Table 1. Our study’s sample size was guided by the sample size of previous studies^4^ ^,^ ^12^ and practical constraints. No *a priori* sample size calculation was performed.

**Table 1:** Demographic characteristics of the participants (N=47).

Variables	
Age (in years) Mean (SD, range)	59.83 (16.59, 30-92)
Time since stroke (in days) Mean (SD, range)	7.4 (4.37, 0-14)
Men N(%)	23 (48.9)
Stroke type: Infarct, hemorrhagic N(%)	38 (80.8), 9 (19.1)
Side of hemiparesis: Right, Left N(%)	20 (42.5), 27 (57.4)
Barthel Index (maximum score: 20) Mean (SD, range)	6.85 (4.5, 0-17)
Functional ambulation classification levels N(%) Level I Level II Level III Level IV Level V	29 (61.7) 14 (29.8) NIL 1 (2.1) 3 (6.4)

### Procedure

We used the behavioral mapping (BM) method to collect data on the patients’ activity patterns. BM is an objective method of observing behavior by an unobtrusive, direct observational method for recording the location of subjects and measuring their activity levels simultaneously^13^. BM is an accepted standard for measuring physical activity for inpatients in the acute stage of stroke and is sensitive to variations in activity level changes^14^ ^-^ ^16^. The observational scheme (recording form) used in this study was based on previous studies^7^ ^,^ ^8^. A physical therapist, trained in the observation method, observed the participants in an unobtrusive manner and collected the data. Participants were observed in the ward on a randomly selected weekday.

Observation was conducted at 10-minute intervals, starting from 8:30 am and ending at 6:00 pm (9.5 hours), which we considered as the most active part of a patient’s day. Fifty-seven observations per patient were recorded, each lasting approximately 1 minute. At each time point, the observer recorded the patient’s activities, persons accompanying patient during activity (family members, nurse, physical therapist, medical professional), and location where the activities took place (hospital room, off ward, bathroom). Patients’ activities were categorized based on the method used by Bernhardt et al.^12^. At each observation, a total of 11 activities could be recorded. These activities were similar to those in previous studies^5^ ^,^ ^12^ ^,^ ^16^ that used BM except for sitting without support, which we excluded as it was uncommon in our setting. If the patient was involved in more than one activity at a time, we recorded only the code of the highest level of activity. For example if the patient was sitting out of bed (therapeutic activity) and talking with the family (non-therapeutic activity), we coded for sitting out of the bed.

Similar to a previous study^3^, we grouped the 11 physical activities into three categories: 1. no activity; 2. non-therapeutic activity (eating, reading/talking/watching television, sitting in bed); 3. therapeutic activities (sitting out of bed, rolling/sitting up, exercising with the therapist, exercising with family (family member helping the patient in doing exercise, standing, walking, independent exercise).

We classified participants’ functional status using the Barthel Index of Activities of Daily Living (BI), one of the most widely used Activities of Daily Living (ADL) scales in stroke rehabilitation^9^ ^,^ ^10^. The BI rates 10 functions on a scale from 0 (fully dependent) to 20 (independent), representing the patient’s ability to carry out daily activities. The following classification system was used in our study: A score of 0-9 indicates severe dependency, a score of 10-19 indicates moderate independence and a score of 20 indicates total independence in ADL^17^. In our study, only two functional groups (severe and moderate) were present as no patient had a score of 20.

### Data analysis

Based on the Kolmogorov-Smirnov test, our data were not normally distributed. Consequently, the values were expressed as the median and IQR or mean and standard deviation (SD). Significance level was set at 0.05 and confidence intervals reported for all analyses. The software used for the analysis was SPSS version 16.0 (SPSS Inc., Chicago, IL, USA).

We documented the activity levels of patients for 9.5 hours (570 minutes, between 8.30am and 6pm). The data were derived from observational units of 1 minute each for every 10 minutes. The past 1-hour activity was derived by multiplying it by a factor of 6. We calculated the median and IQR of proportion of time spent in each activity level, namely no activity, non-therapeutic activity, and therapeutic activity, along with the people present during the activity and the locations of the activity.

Using the Mann-Whitney U test, we analyzed the difference between activity level and functional status by comparing mean proportion of time spent in each activity category (no activity, non-therapeutic activity, and therapeutic activity) and patient group, categorized as moderate or severe functional limitations.

To study the association between the presence of family members and activity level, we compared the mean proportion of time spent inactive (no activity), in therapeutic and non- therapeutic activities, with the mean proportion of time spent with family using Pearson’s correlation coefficient as both variables are continuous (interval). The correlation coefficient (*r*) was interpreted as low (0.26-0.49), moderate (0.50-0.69), high (0.70-0.89), or very high (0.90-1.00)^18^.

## Results

### Physical activity, people present, and location of activity

The median proportion of time spent in each activity is reported in Table 2. Participants were inactive for 19% (IQR 12-36%) of the time during the day. They spent 46% (IQR 31-55%) of their time in therapeutic activities and 31% (IQR 22-34%) in non-therapeutic activities. Patients were alone for 15% (IQR 10-19%) of the time and were accompanied by family 50% (IQR 45-55%) of the time. They spent 78% (IQR 71-86%) of the time in their personal room (Table 2).

**Table 2:** Proportion of time spent in different activities, locations, alone, or with others.

**Activities (N=47)**	**Median % of time spent in a day (IQR)**
No activity (in bed)	19.0 (12-36)
Leisure (read/talk/watch television)	7.1 (4-10)
Eating	2.8 (2-4)
Sit in bed	19.2 (9-24)
Sit out of bed	13.7 (5-21)
Exercise with family	2.9 (0-5)
Roll/sit up	3.0 (2-5)
Exercise with therapist	7.0 (3-9)
Standing activities	7.3 (3-9)
Walking	7.0 (0-16)
Independent exercise	3.0 (0-5)
**Locations**	
Bathroom	2.7 (0-5)
Off ward	9.3 (0-10)
Personal room	78.0 (71-86)
**People present**	
Alone	15.0 (10-19)
Family	50.0 (45-55)
Nurse	12.4 (10-16)
Medical Professional (Physician)	6.6 (7-10)
Physical therapist	17.0 (10-19)
	

### Physical activity, functional status, and family presence

As anticipated, patients classified as severely dependent in daily activities spent a high proportion of time inactive compared with those who were moderately dependent (mean difference 19.47%; 95% CI, 12.33 to 26.66, p=0.00). They spent less time in therapeutic activities (mean difference 18.7%; 95% CI, 26.81 to 10.60, p=0.00). There was no significant between-group difference in the amount of time spent in non-therapeutic activities. The family’s presence was inversely associated with no activity (*r*=–0.71, 95% CI 0.53 to 0.82, p=0.00) but directly associated with therapeutic activity (*r*=0.60, 95% CI 0.13 to 0.61, p=0.00) and non-therapeutic activity (*r*=0.40, 95% CI 0.38 to 0.76, p=0.00) (Table 3).

**Table 3:** Comparison of proportion of time spent based on functional status.

Activity Category	Functional status	N	Mean (SD) Time spent in a day (%)	Mean Difference	p	95% CI of the Difference		
						Lower	Upper
No activity	Severe	34	30.5 (18.4)	19.5	.00	12.3	26.6
	Moderate	13	11.0 (5.8)				
Nontherapeutic activity	Severe	34	28.6 (10.8)	-1.0	.73	-6.8	4.8
	Moderate	13	29.6 (7.9)				
Therapeutic activity	Severe	34	40.1 (13.1)	-18.7	.00	-26.8	-10.6
	Moderate	13	58.8 (11.6)				

## Discussion

Patients admitted to the medical ward of Indian hospitals within 14 days of stroke spent less time being inactive and alone and more time with family participating in therapeutic activities. This result is in contrast with previous studies’ findings, which reported that patients were alone and inactive for most of their day^3^ ^-^ ^6^. We also found that family presence with the patient and patient’s moderate dependence in daily activities were positively associated with their activity levels.

Bernhardt et al.^9^ suggested that it would help to compare different models of care and replicate models of care that promote better outcomes for patients, thereby contributing toward development of agreed standards of care. The physical activity patterns of the participants of this study are distinct from the high levels of inactivity reported by previous studies conducted in Australia^9^, UK, and Europe^4^. In our study, even those stroke patients classified as severe spent significantly less time (35% of the day) in no activity when compared to the results reported in studies done in Australia (95% of the day)^12^ and Europe (72% of the day)^7^. This might be due to the increased family presence (50% of the day) with the patients observed in our study. These findings are supported by previous studies that evaluated the family’s role in stroke rehabilitation^19^ ^,^ ^20^. These studies found individuals with moderate and severe stroke who had high levels of social support attained a significantly better and progressively improved functional status than those with less support^20^ ^,^ ^21^. Hence, efforts at reducing the patients’ time alone by increasing family presence could potentially improve their activity levels, especially in patients with lower functional status. Further, other related factors such as extended therapy time (approximately 90 minutes/day) and no restrictions to manual handling of patients by physical therapists have been shown to be associated with increased activity level^5^ ^,^ ^9^. However, based on this study’s findings, it is not possible to explain the role of these factors in contributing to the patient’s physical activity level.

This study has a few limitations. Our study results may have been confounded by factors such as observer bias, patient’s age, psychological status, and time since stroke. We did not analyze these influences due to our limited sample size. Considering the size of the differences observed between activity categories, however, these factors may not have had a major influence on the study results.

### Clinical implications

Increased time spent in bed during the early phase after stroke is associated with poor functional outcome and recovery^8^. Time spent in therapeutic activities, such as standing and walking in the early phase after stroke, have been found to positively influence recovery^1^ ^,^ ^2^. Therefore, hospital policies that promote more therapeutic activities and increased opportunity for socialization and therapy time could be beneficial, ensuring maximum family presence with patients early after stroke and educating the family about the need for physical activity. This may lead to increased time being active and less time alone and on non-therapeutic activities such as sitting in bed.

## Conclusion

The characteristics of the stroke rehabilitation setting can influence the physical activity levels of patients after acute stroke. Patients admitted to Indian hospitals are active and spend more time with family participating in therapeutic activities. The presence of family members with the patient during hospital stay may be a significant resource for encouraging patients to be more active.

## References

[1] Askim T, Bernhardt J, Salvesen O, Indredavik B (2014). Physical activity early after stroke and its association to functional outcome 3 months later. J Stroke Cerebrovasc Dis.

[2] West T, Churilov L, Bernhardt J. (2013). Early physical activity and discharge destination after stroke: a comparison of acute and comprehensive stroke unit care. Rehabil Res Pract.

[3] West T, Bernhardt J (2012). Physical activity in hospitalised stroke patients. Stroke Res Treat.

[4] Wellwood I, Langhorne P, McKevitt C, Bernhardt J, Rudd AG, Wolfe CDA (2009). An observational study of acute stroke care in four countries: the European registers of stroke study. Cerebrovasc Dis.

[5] De Weerdt W, Selz B, Nuyens G, Staes F, Swinnen D, van de Winckel A (2000). Time use of stroke patients in an intensive rehabilitation unit: a comparison between a Belgian and a Swiss setting. Disabil Rehabil.

[6] Bernhardt J, Chitravas N, Meslo IL, Thrift AG, Indredavik B (2008). Not all stroke units are the same: a comparison of physical activity patterns in Melbourne, Australia, and Trondheim, Norway. Stroke J Cereb Circ..

[7] Hokstad A, Indredavik B, Bernhardt J, Ihle-Hansen H, Salvesen Ø, Seljeseth YM (2015). Hospital differences in motor activity early after stroke: a comparison of 11 Norwegian stroke units. J Stroke Cerebrovasc Dis.

[8] Langhorne P, de Villiers L, Pandian JD (2012). Applicability of stroke-unit care to low-income and middle-income countries. Lancet Neurol.

[9] Bernhardt J, Chitravas N, Meslo IL, Thrift AG, Indredavik B (2008). Not all stroke units are the same: a comparison of physical activity patterns in Melbourne, Australia, and Trondheim, Norway. Stroke J Cereb Circ..

[10] Wasay M, Khatri IA, Kaul S (2014). Stroke in South Asian countries. Nat Rev Neurol.

[11] Aho K, Harmsen P, Hatano S, Marquardsen J, Smirnov VE, Strasser T (1980). Cerebrovascular disease in the community: results of a WHO Collaborative Study. Bull World Health Organ.

[12] Bernhardt J, Dewey H, Thrift A, Donnan G (2004). Inactive and alone: physical activity within the first 14 days of acute stroke unit care. Stroke J Cereb Circ..

[13] Cosco NG, Moore RC, Islam MZ (2010). Behavior mapping: a method for linking preschool physical activity and outdoor design. Med Sci Sports Exerc.

[14] Fini NA, Holland AE, Keating J, Simek J, Bernhardt J (2015). How is physical activity monitored in people following stroke?. Disabil Rehabil.

[15] Stephens MA, Norris-Baker C, Willems EP (1983). Patient behavior monitoring through self-reports. Arch Phys Med Rehabil.

[16] Bernhardt J, Chan J, Nicola I, Collier JM (2007). Little therapy, little physical activity: rehabilitation within the first 14 days of organized stroke unit care. J Rehabil Med.

[17] Vermeulen CJ, Buijck BI, van der Stegen JC, van Eijk MS, Koopmans RT, Hafsteinsdóttir TB (2013). Time use of stroke patients with stroke admitted for rehabilitation in Skilled Nursing Facilities. Rehabil Nurs.

[18] Cohen J (2013). Statistical power analysis for the behavioral sciences.

[19] Maeshima S, Ueyoshi A, Osawa A, Ishida K, Kunimoto K, Shimamoto Y (2003). Mobility and muscle strength contralateral to hemiplegia from stroke: benefit from self-training with family support. Am J Phys Med Rehabil.

[20] Tsouna-Hadjis E, Vemmos KN, Zakopoulos N, Stamatelopoulos S (2000). First-stroke recovery process: the role of family social support. Arch Phys Med Rehabil.

[21] Tyson SF, Burton L, McGovern A (2016). The effect of a structured programme to increase patient activity during inpatient stroke rehabilitation: A Phase I cohort study. Clin Rehabil.

